# Supplementary material for: DMRforPairs: identifying Differentially Methylated Regions between unique samples using array based methylation profiles
Source: BMC Bioinformatics. 2014 May 15;15:141. doi: 10.1186/1471-2105-15-141 (PMC4046028; doi:10.1186/1471-2105-15-141)
Supplement: Additional file 2 — DMRforPairs output for the comparison of A431-MCF7 and NA17018-NA17105. Please start from the HTML files in each folder. Available via the BMC Bioinformatics website. [file 1471-2105-15-141-S2.zip › 1394847754114233_MOESM2_ESM/A431_MCF7/figures/29994.pdf]

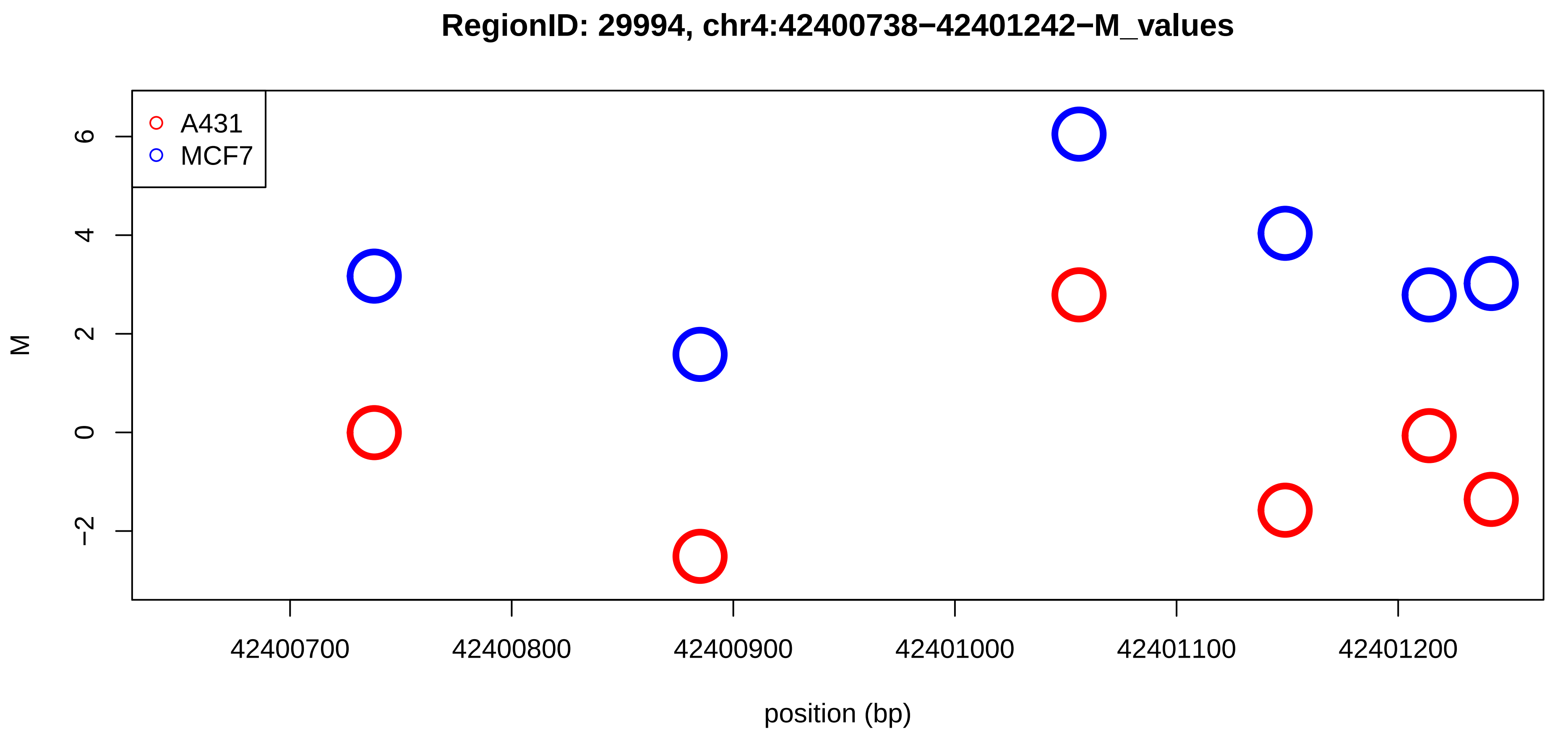

RegionID: 29994, chr4:42400738−42401242−M_values
A431
MCF7
M
−2
0
2
4
6
42400700
42400800
42400900
42401000
42401100
42401200
position (bp)

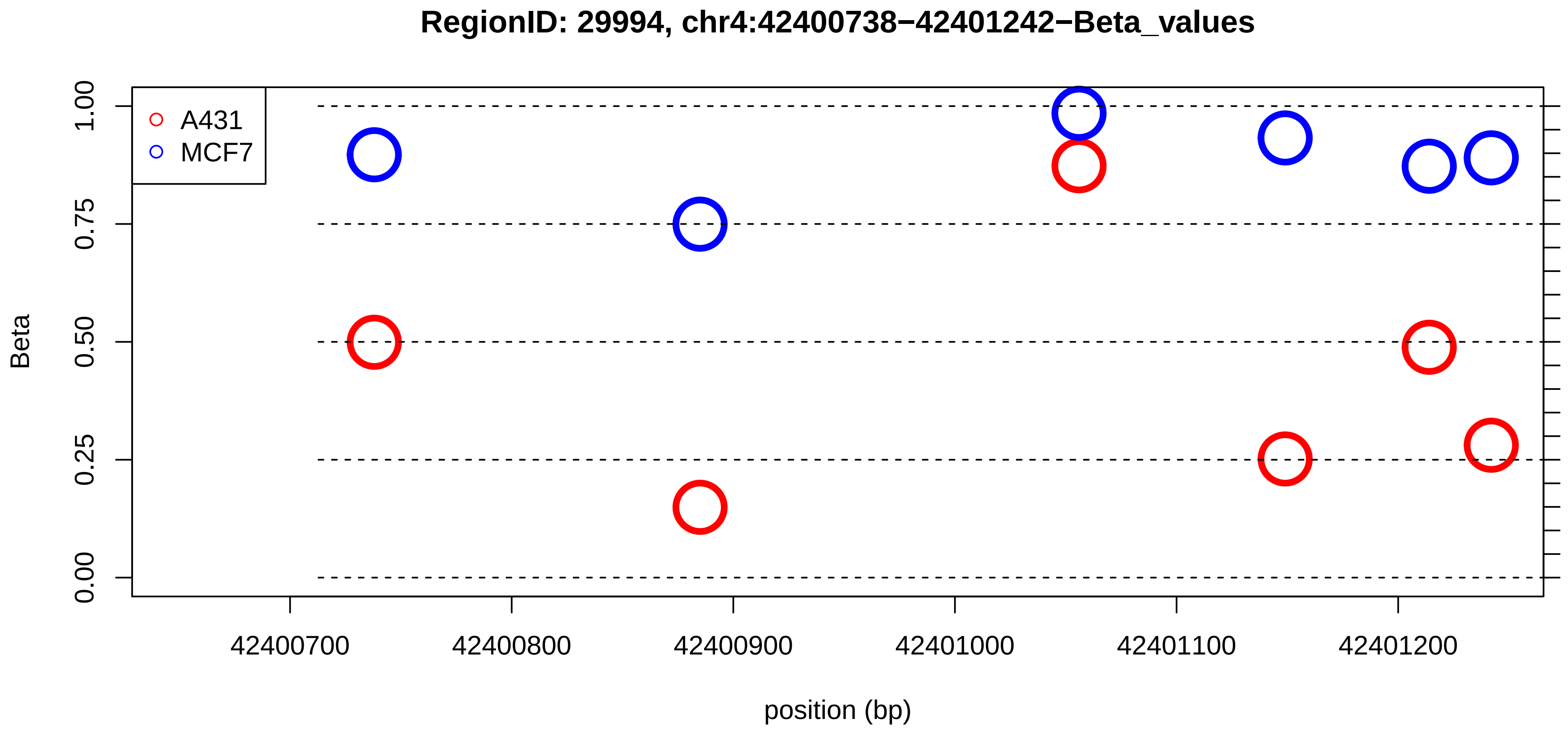

RegionID: 29994, chr4:42400738−42401242−Beta_values
A431
MCF7
Beta
0.00
0.25
0.50
0.75
1.00
42400700
42400800
42400900
42401000
42401100
42401200
position (bp)

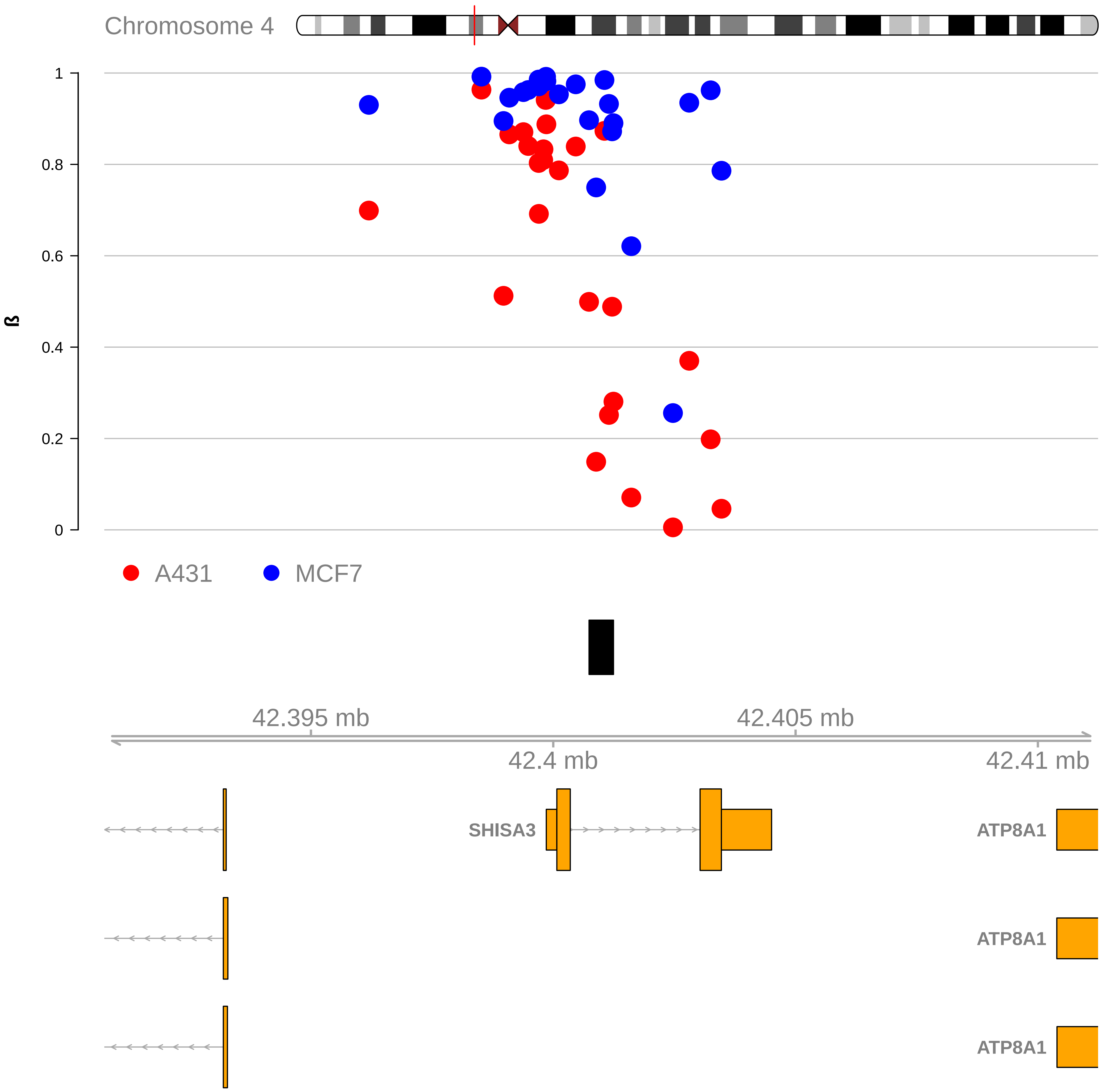

Chromosome 4
1
0.8
0.6
0.4
0.2
0
ß
A431
MCF7
42.395 mb
42.405 mb
42.4 mb
42.41 mb
SHISA3
ATP8A1
ATP8A1
ATP8A1
